# High-Temperature Formability and Friction Regulation Mechanism of TA17 Titanium Alloy with Typical Microstructures

**DOI:** 10.3390/ma19112260

**Published:** 2026-05-27

**Authors:** Bin Yan, Guocheng Zhang, Xiaoli Liu, Yidi Yang

**Affiliations:** 1 School of Mechanical and Aviation Manufacturing Engineering, Anyang Institute of Technology, Anyang 455000, China; yanb_1988@163.com; 2 College of Mechanical and Equipment Engineering, Hebei University of Engineering, Handan 056038, China; liuxiaoli01206332@163.com (X.L.); yidi_yang0307@163.com (Y.Y.); 3 Key Laboratory of Intelligent Industrial Equipment Technology of Hebei Province, Handan 056038, China

**Keywords:** friction, TA17, microstructures, formability

## Abstract

This study aims to clarify the effects of initial microstructure and friction coefficient on the high-temperature formability of TA17 titanium alloy. Three typical microstructures were prepared via different annealing processes to provide theoretical and experimental support for hot forming process optimization. Existing studies on TA17 titanium alloy mainly focus on its room-temperature mechanical properties and corrosion resistance, while quantitative investigations on the influence of different initial microstructures on its high-temperature forming limit are still scarce. Moreover, the temperature evolution characteristics of the friction coefficient for TA17 alloy and its quantitative influence on deep-drawing forming limit remain unclear, and few studies have considered the effect of initial microstructure and friction coefficient on the high-temperature formability of titanium alloys. Combined with high-temperature tensile tests, high-temperature friction- wear tests and finite element (FE) simulation, the effects of microstructure characteristics and friction coefficient on the high-temperature formability of TA17 alloy were systematically investigated. The results show that the equiaxed microstructure obtained by annealing at 850 °C for 2 h exhibits the best high-temperature plasticity at 900 °C and strain rate of 0.01 s^−1^, while the plasticity of Widmanstätten and bimodal structures is significantly reduced. The high-temperature friction coefficient of TA17 alloy decreases sharply with increasing temperature, dropping from 0.56 at 650 °C to 0.19 at 800 °C, and can be stably controlled below 0.2 in the optimal forming temperature range of 800–900 °C. The friction coefficient has a remarkable influence on the deep-drawing forming limit: as the friction coefficient increases from 0.05 to 0.2, the limit principal strain and limit punch stroke decrease accordingly. This study reveals that fine equiaxed microstructure and low friction coefficient can enhance the high-temperature forming formability of TA17 titanium alloy. In actual industrial hot forming processes, it is recommended to use TA17 alloy with equiaxed microstructure and control the friction coefficient below 0.15 by using high-temperature lubricants, which can effectively improve the forming quality of complex aerospace structural components.

## 1. Introduction

TA17 (Ti-4Al-2V) [1], a typical medium-strength near-α titanium alloy, integrates outstanding room-temperature strength and toughness, corrosion resistance, and medium–high-temperature plastic forming ability. Additionally, it offers the benefits of reasonable processing costs and high process adaptability, and is extensively utilized in the fabrication of complex structural components for high-end equipment in the aerospace, marine, and military sectors. Owing to their pronounced resistance to plastic deformation and substantial elastic recovery at ambient temperature, titanium alloy sheet parts are typically fabricated using hot-forming processes [2,3]. Such titanium alloy components are predominantly fabricated via plastic forming processes, including hot forging and high-temperature deep drawing. The forming quality directly dictates the service performance and service life of the components. Consequently, investigating the regulatory laws governing the high-temperature forming performance of TA17 titanium alloy holds significant practical importance for its engineering application.

Microstructure serves as the core factor that determines the high-temperature plastic deformation behavior of titanium alloys. Variations in parameters of hot working and heat treatment processes can lead to the formation of different typical microstructures, including equiaxed, Widmanstätten, and bimodal structure, by regulating dynamic recrystallization, phase transformation, and grain growth [4]. Microstructures with different morphologies exhibit significant disparities in grain boundary characteristics, phase composition, and distribution. These disparities directly alter high-temperature deformation mechanisms such as dislocation movement and grain boundary sliding [5], thereby exerting a decisive influence on the flow stress, plasticity index, and formability limit of titanium alloys. Consequently, optimizing the high-temperature forming performance by controlling the microstructure through process regulation has emerged as a research focus in the field of titanium alloy forming [6,7,8].

Currently, extensive studies have been conducted on the microstructure control and high-temperature forming performance of titanium alloys [9,10]. Scholars have verified the regulatory effects of annealing temperature, holding time and cooling rate on the phase composition and grain size of titanium alloys, and revealed the correlation between microstructure and high-temperature mechanical properties of typical alloys such as TC4 and TA15. However, for TA17 titanium alloy, there are still no quantitative investigations on the effects of different initial microstructures on its high-temperature forming limit, and the underlying deformation mechanisms have not been systematically elucidated. Meanwhile, high-temperature friction between sheet and die is another critical factor affecting metal hot forming quality. It changes the stress distribution in the deformation zone, aggravates forming defects such as wrinkling and cracking, and further reduces the forming limit [11]. Although the friction behavior of TC4 and TA15 alloys at elevated temperatures has been reported [12,13,14], the temperature evolution characteristics of friction coefficient for TA17 alloy and its quantitative influence on deep-drawing forming limit remain unclear. More importantly, few studies have considered the effect of initial microstructure and friction coefficient on the high-temperature formability of titanium alloys, which limits the precise optimization of hot forming processes.

In this work, industrial hot-rolled TA17 titanium alloy billets were used as the research material. Three distinct annealing processes were employed to prepare three typical microstructures. The effects of initial microstructure on the high-temperature mechanical properties, forming limits, and fracture mechanisms were systematically investigated through uniaxial tensile tests conducted at 900 °C. The temperature dependence of the high-temperature friction coefficient was characterized via friction-wear tests, and the quantitative influence of friction coefficient on the forming limit of circular cup deep drawing was analyzed using finite element (FE) simulation. Finally, the effect of microstructure and friction coefficient was revealed, which provides experimental data and theoretical support for the optimal design of TA17 hot forming process.

## 2. Materials and Methods

### 2.1. Experimental Materials

The raw materials adopted in this experiment were industrial-grade TA17 titanium alloy hot-rolled billets. The chemical composition is presented in Table 1. The principal alloying elements are 4.3 wt.% aluminum and 2.1 wt.% vanadium. The mass fractions of impurity elements, including Fe, Si, O, N, H, and C, were rigorously controlled below 0.25 wt.%, with titanium as the balance. The β transus of this bar is determined as approximately 910~920 °C by the metallographic method.

### 2.2. Heat Treatment Process

To obtain three distinct microstructures, three sets of annealing processes were employed [15], as presented in Table 2. Subsequent to the annealing tests, the microstructure of the titanium alloy was examined using an optical microscope. The metallographic specimens were ground sequentially using silicon carbide sandpapers with grit sizes of 180#, 400#, 800#, 1200# and 2000#. Each grinding step was performed at a 90° rotation relative to the previous direction until all scratches from the prior grit were completely eliminated, with running water used for cooling throughout the process. After each grinding step, the specimens were ultrasonically cleaned in anhydrous ethanol for 3 min to remove residual abrasive particles and debris. Subsequently, the specimens were polished with Al_2_O_3_ polishing powder, resulting in a specimen surface free of obvious scratches. After polishing, the specimens were again ultrasonically cleaned in anhydrous ethanol for 5 min and then dried with cold air immediately. Subsequently, they were etched with a reagent having a volume ratio of HF:HNO_3_:H_2_O of 10:15:75 for 5 to 10 s, and finally cleaned with alcohol and dried for future use. Ultimately, the optical microscope observation was conducted.

### 2.3. Hot Tensile Test

**Figure 1:**
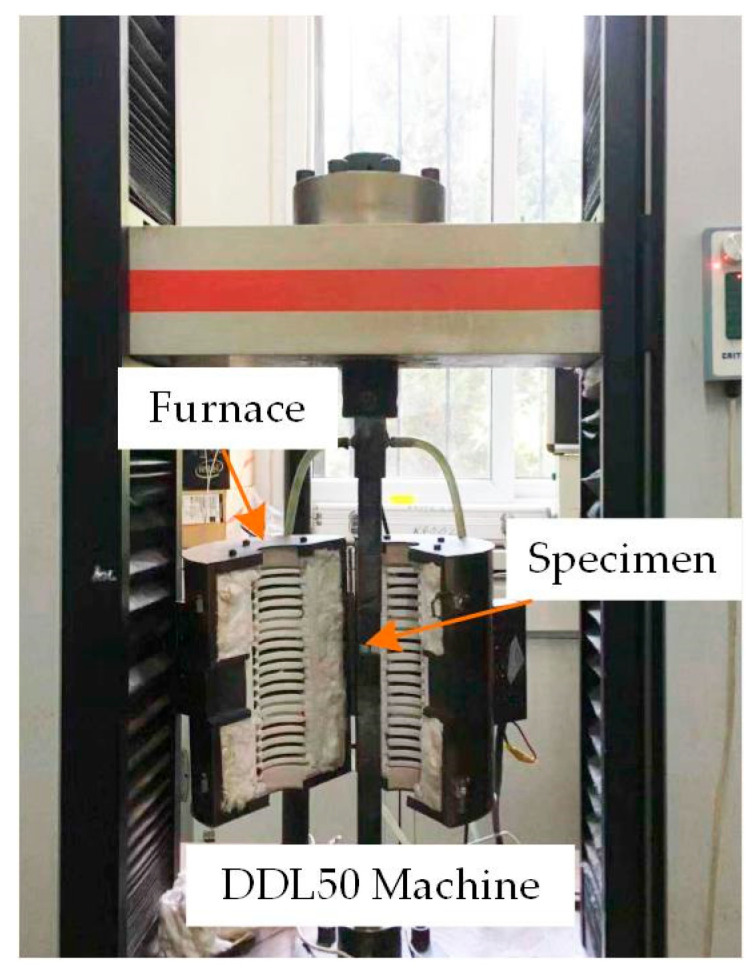
DDL50 uniaxial tensile experimental equipment.

To acquire the high-temperature mechanical properties of TA17 titanium alloy, tensile tests at a high temperature were performed on the annealed specimens. Each type of specimen was subjected to testing three times to minimize experimental errors. As depicted in Figure 1, high-temperature tensile tests were conducted on a DDL50 uniaxial tensile experimental equipment (Changchun Mechanical Research Institute, Changchun, China) at 900 °C and a strain rate of 0.01 s^−1^ to obtain the flow stress curves [16]. Following cleaning treatment of the fracture surfaces of the high-temperature tensile specimens, the fracture morphology was characterized via a Zeiss Sigma 300 scanning electron microscope (SEM, Zeiss, Oberkochen, Germany), and the corresponding fracture mechanism was analyzed.

### 2.4. Friction Coefficient Test

**Figure 2:**
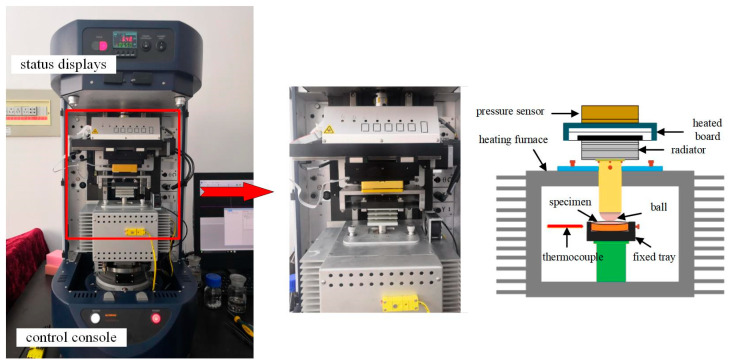
Friction wear testing machine.

To explore the effect of friction coefficient on the formability of titanium alloy sheets, the evolution law of the friction coefficient of titanium alloy sheets with temperature was first characterized, so as to define the value range of the friction coefficient at high temperatures. Consequently, high-temperature friction tests were performed on titanium alloy plates. As depicted in Figure 2, the friction tests were conducted on a Bruker UMT-Tribolab tribometer (Bruker Nano Inc., Berlin, Germany). The primary characteristic of this tribometer is that it is equipped with an independent high-temperature component. Its high-temperature chamber is replaceable, and the built-in thermocouple exhibits extremely high sensitivity, with a temperature control accuracy reaching up to 0.01 °C. Furthermore, the top of the upper test ball clamping device component is furnished with a high-precision pressure sensor, which can accurately control the test load for friction force measurement. Its data acquisition module comprises 8 data acquisition channels and a 16-bit data acquisition system, with a maximum sampling frequency of up to 200 kHz. This device is capable of meeting the requirements of diverse high-temperature friction and wear tests and exhibits good repeatability, thereby fulfilling the demands of friction and wear tests under multiple conditions. The TA17 titanium alloy specimens used in the experiment measured 15 mm × 15 mm × 5 mm, with their wear surfaces untreated to reflect actual production conditions. The tests were conducted at a rotational speed of 560 rpm for 30 min in an air atmosphere. Three parallel tests were performed, yielding a standard deviation of the friction coefficient of less than 0.03. The small balls with a diameter of 10 mm, which are selected for the wear tests, are fabricated from GCr15 [14], and their chemical composition is presented in Table 3. This material possesses excellent comprehensive performance, high hardenability, uniform hardness subsequent to heat treatment, good dimensional stability, high contact fatigue strength, and strong corrosion resistance, rendering it highly suitable for high-temperature friction and wear test research. Although the ball-on-plate configuration differs from the actual sheet–die contact in deep drawing, it is a standard method for comparative evaluation of high-temperature friction properties. The contact pressure in the test is close to the maximum contact pressure in hot deep drawing. The GCr15 steel used in the test is a common die material for titanium alloy hot forming.

**Figure 3:**
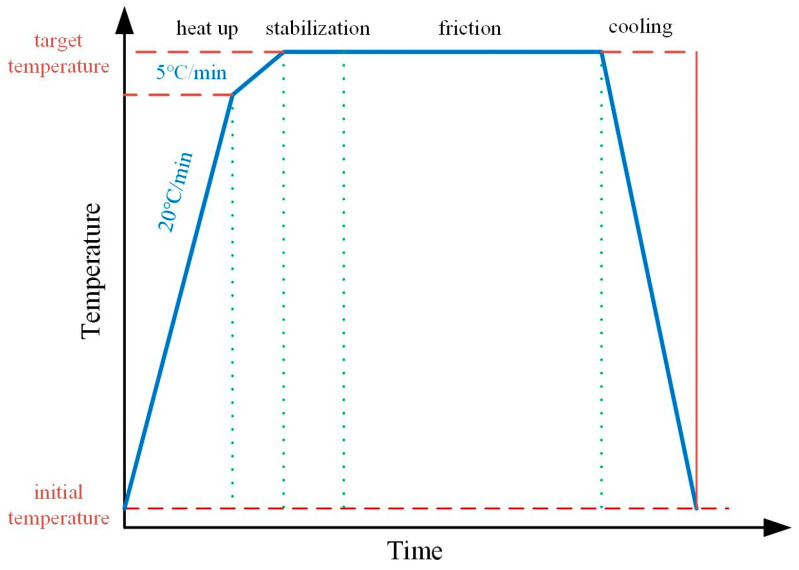
High-temperature friction test process.

The operation process of the UMT-type friction and wear testing machine is controlled by programming via the integrated TriboScript software for system control and data analysis. Consequently, the test process is translated into a segmented program and input into the software. As depicted in Figure 3, to ensure uniform heating of the sample and eliminate the influence of temperature delay, the following heating procedure was adopted for the testing machine: the heating rate was set at 20 °C/min when the temperature was 40 °C below the target temperature, and then reduced to 5 °C/min to ensure that the temperature could accurately reach the preset value. Upon reaching the target temperature, the sample was held for 10 min to achieve thermal stability. Subsequently, the friction test was performed under the pre-set load, time and frequency for a duration of 30 min. After the test was completed, the sample was furnace-cooled to room temperature together with the testing machine.

## 3. Results and Discussion

### 3.1. Microstructure

**Figure 4:**
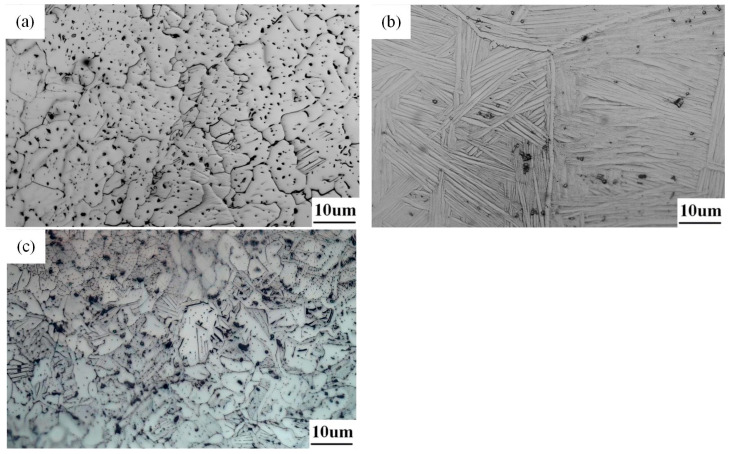
Microstructure: (**a**) equiaxed structure (A1); (**b**) Widmanstätten structure (A2); (**c**) bimodal structure (A3).

Three typical titanium alloy microstructures were acquired through annealing using different processes, as depicted in Figure 4. The quantitative analysis of microstructural parameters was performed using Image-Pro Plus 6.0 software. At least five optical micrographs were analyzed for each sample. Grain size was measured by the linear intercept method, and phase fractions were determined by threshold segmentation.

Figure 4a depicts a typical fine equiaxed α titanium alloy microstructure [17]. This microstructure is predominantly composed of a substantial quantity of nearly equiaxed α phases (α), featuring a grain size of approximately 5~8 μm. The grain boundaries are distinct and uniformly distributed, exhibiting no evident grain growth or preferred orientation. Black residual β phases (β) with a volume fraction of approximately 10–15% are dispersed at the grain boundaries and among the grains. No apparent second-phase segregation or macroscopic defects were detected. During the hot working procedure, dynamic recrystallization (DRX) facilitates the refinement and equiaxialization of the α grains, and the subsequent annealing further alleviates the processing stress, resulting in the uniform dispersion of the residual β phases [18,19].

Figure 4b depicts a typical lamellar Widmanstätten microstructure of a titanium alloy. This microstructure consists of large primary β grains (β) [20], with sizes ranging from 50 to 100 μm. The grain boundaries are clearly delineated by thick black lines, suggesting evident grain anisotropy. Inside the primary β grains, numerous lamellar α phases (α) have grown, with a thickness of approximately 0.5 to 1 μm. Lamellar bundles with different orientations create a “spike-like” Widmanstätten morphology, and a small quantity of black β phase persists at the interfaces between lamellae and at the grain boundaries. This microstructure is typically achieved through hot working in the β single-phase region, followed by air cooling or furnace cooling. Hot working in the β region leads to significant growth of the primary β grains. During the slow cooling process, the α phase precipitates along the β grain boundaries and specific crystal planes within the grains, resulting in the formation of a lamellar Widmanstätten structure. The coarse β grain boundaries and continuous lamellar α colonies will hinder coordinated deformation between grains, potentially leading to stress concentration at the lamellar interfaces, which is detrimental to high-temperature plasticity [21].

Figure 4c depicts a typical bimodal structure titanium alloy microstructure (equiaxed α + lamellar α/β mixed structure), which is composed of two parts in a coordinated manner [22]. One is the finely dispersed equiaxed α phase (white, with a size of approximately 3~6 μm), having a volume fraction of around 30~40%. The other is the lamellar α/β mixed structure (gray/black) that fills the spaces between the equiaxed α phases. The lamellar scale of this structure is significantly finer than that shown in Figure 4b. The equiaxed α phases are uniformly incorporated within the lamellar matrix, without evident grain boundary segregation or microstructural inhomogeneity, and the overall microstructure demonstrates a favorable coordinated distribution characteristic. This microstructure is typically obtained through hot working in the near-β zone, followed by an annealing treatment. Hot working in the near-β zone not only preserves some untransformed equiaxed α phases but also induces the precipitation of fine lamellar α from the matrix β phase during cooling, ultimately resulting in a bimodal structure consisting of equiaxed α and lamellar α/β [23].

### 3.2. High-Temperature Forming Performance

**Figure 5:**
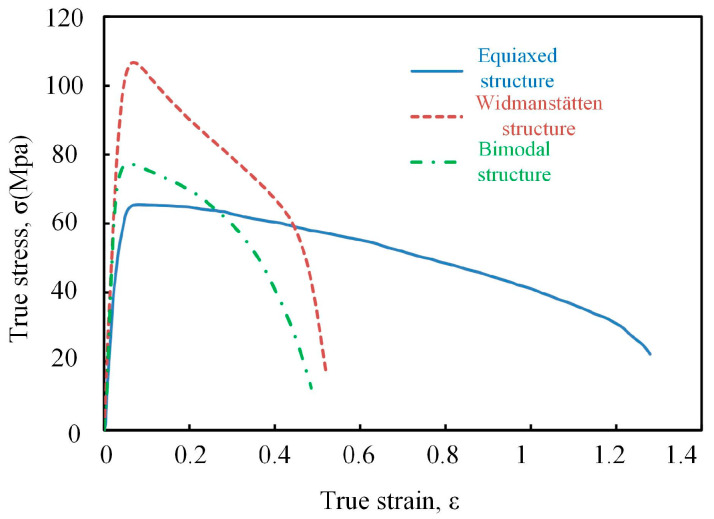
Tensile curves of different microstructures at 900 °C and a strain rate of 0.01 s^−1^.

**Figure 6:**
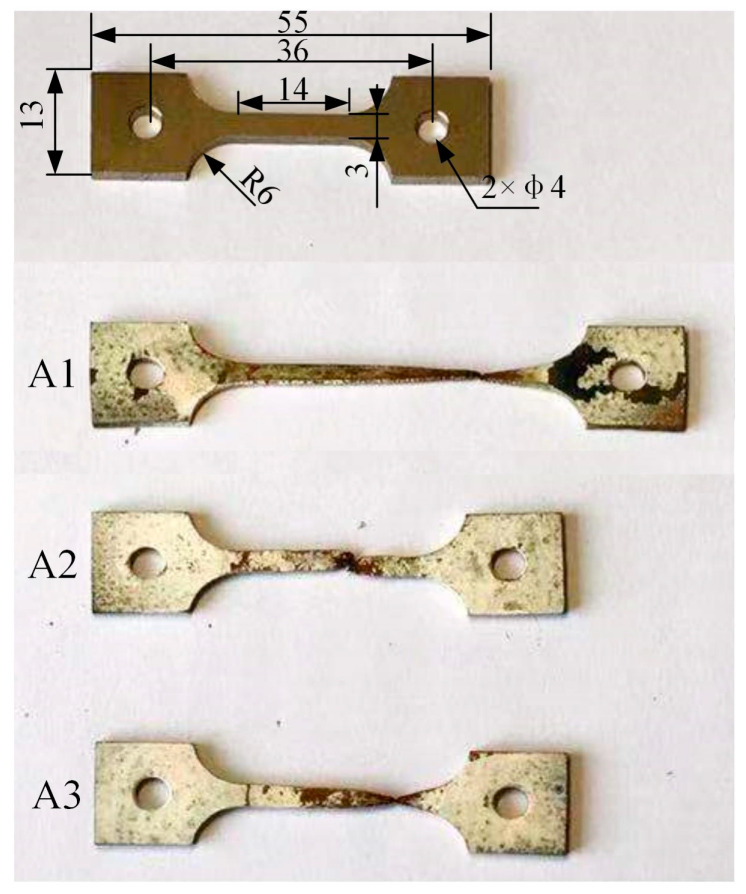
High-temperature tensile specimens (unit: mm).

To investigate the influence of diverse microstructures on the forming limit, equiaxed, Widmanstätten, and bimodal structure were chosen for examination. The mechanical property curves of TA17 titanium alloy with these three microstructures, which were acquired from tensile tests conducted at 900 °C and a strain rate of 0.01 s^−1^, are presented in Figure 5 and Table 4. The high-temperature tensile specimens are depicted in Figure 6.

**Figure 7:**
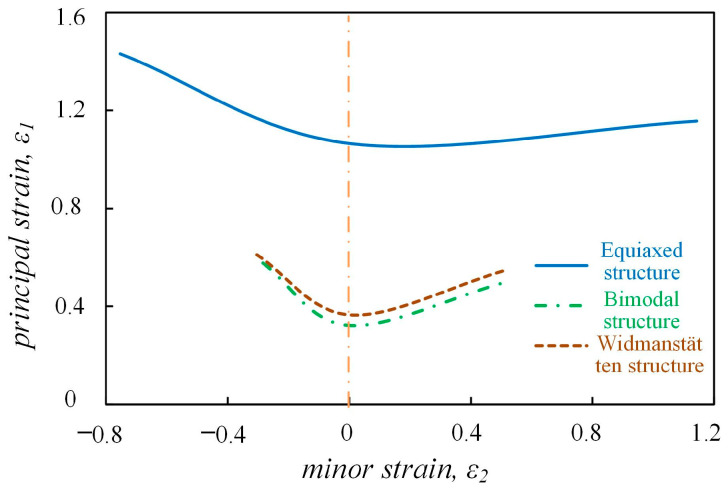
High-temperature forming limits under three different microstructures.

Utilizing the ABAQUS 6.14 finite element software and implementing the previous studies [24], the forming limits of TA17 under the three microstructures were ascertained based on the experimental results presented in Figure 5. The obtained high-temperature forming limits under the three different microstructures are illustrated in Figure 7. It is evident from the figure that the forming limits of different microstructures exhibit significant variations, and the forming limit of the equiaxed microstructure is the most favorable.

### 3.3. Fracture Morphology

**Figure 8:**
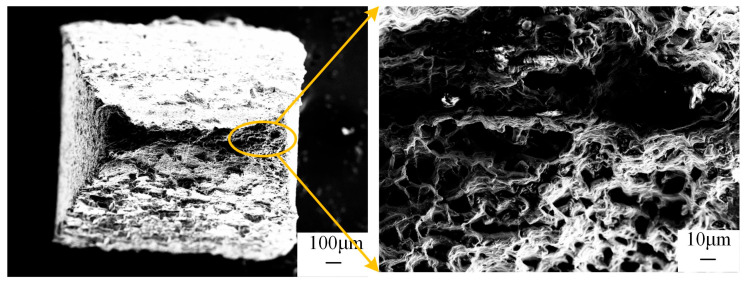
High-temperature tensile fracture morphology.

The fracture morphology of the uniaxial tensile specimen’s tensile fracture was examined. The overall morphology of the fracture surface at 100× magnification and the local morphology at 1000× magnification are presented in Figure 8. The macroscopic fracture surface exhibits typical ductile fracture characteristics, featuring a rough and uneven surface along with distinct plastic necking marks. The overall fracture morphology is fibrous, lacking clear cleavage planes or river-like cleavage features, suggesting that a substantial amount of plastic deformation occurred in the titanium alloy during high-temperature tensile testing [25].

### 3.4. High-Temperature Friction Behavior

**Figure 9:**
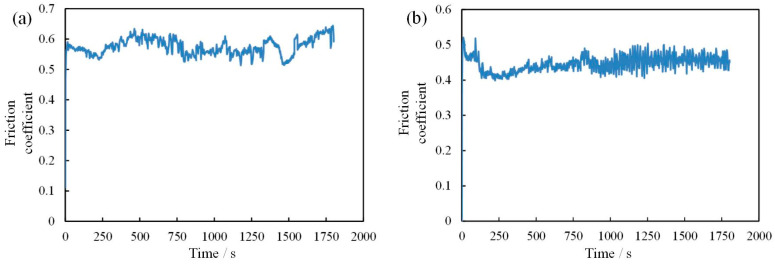

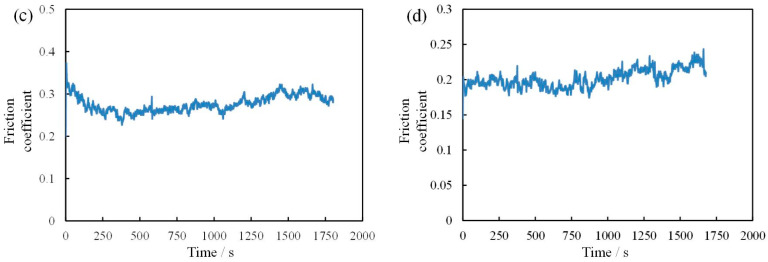
Friction coefficient curves at different temperatures: (**a**) 650 °C; (**b**) 700 °C; (**c**) 750 °C; (**d**) 800 °C.

**Figure 10:**
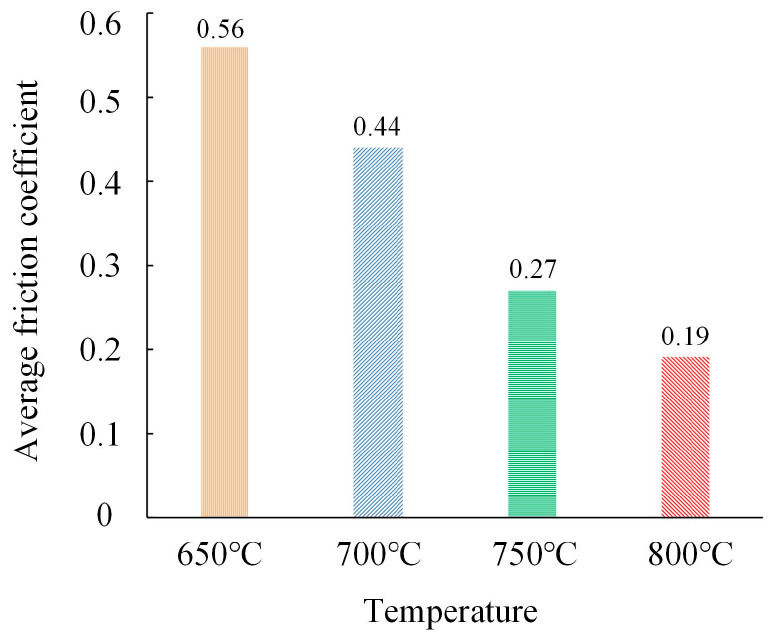
Average friction coefficient at different temperatures.

The friction tests were performed on samples with equiaxed microstructure obtained by A1 annealing. Owing to the limitations of the test equipment and conditions, the high-temperature friction test was carried out at 650–800 °C, as depicted in Figure 9. The variation curves of the friction coefficient with respect to time under different temperatures were acquired. The average friction coefficients at different temperatures were calculated and are presented in Figure 10. It is evident that the friction coefficient decreases with increasing temperature. Lu Haifeng [26] investigated the friction and wear performance of TC4-DT titanium alloy at different temperatures. They found that as the temperature rises, the friction coefficient of TC4-DT titanium alloy continuously declines, and the wear degree is significantly reduced, with a reduction of 83.7%. The main reason for the decrease in friction coefficient with increasing temperature is the formation of a continuous and dense TiO_2_-Al_2_O_3_ composite oxide film on the contact surface. At high temperatures, titanium alloy is easily oxidized, and the oxide film has good lubrication properties, which can isolate the direct contact between the titanium alloy and the steel ball, thus reducing adhesive wear and friction coefficient [14]. As the temperature increases, the oxide film becomes thicker and more continuous, and its lubrication effect is more significant. Therefore, when forming at a temperature higher than 800 °C, the friction coefficient is less than 0.2. Consequently, the friction coefficients of 0.05, 0.1, 0.15, and 0.2 were selected to examine the influence of the friction coefficient on the forming performance at 900 °C. This inference needs to be verified by further experiments at higher temperatures.

**Figure 11:**
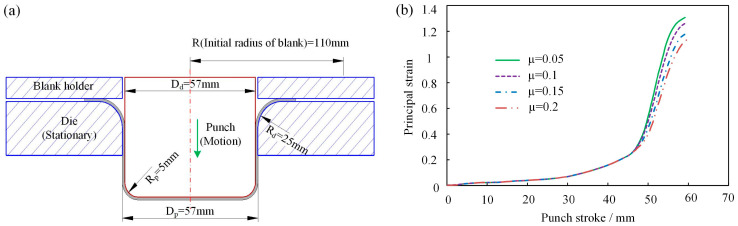
(**a**) Schematic diagram; (**b**) the principal strain under different friction coefficients.

Finite element simulations of cup drawing [24] were carried out with friction coefficients of 0.05, 0.1, 0.15, and 0.2. Given that the friction force increases with the increase in forming speed, and the metal forming rate is considerably lower than the speed of the friction test [27], the friction coefficient in the forming test is lower than that obtained in the friction test. Finite element simulation of circular cup drawing was carried out using ABAQUS. The geometric model includes punch, die, blank holder and sheet. The shape and dimension of specimen and tools in cup drawing test is shown in Figure 11a. The blank holder force is 10 kN. The sheet is a circular blank with diameter 110 mm and thickness 2 mm. Punch, die and blank holder are set as rigid bodies, and the sheet is a deformable body. Boundary conditions: punch moves downward at 1 mm/s; die is fully fixed; blank holder is only allowed to move along *Z*-axis. The curves depicting the relationship between the principal strain at the failure point and the stroke depth during the drawing process are presented in Figure 11b. As can be observed from the figure, the variation in the friction coefficient influences the evolution of the principal strain at the failure point. When the punch stroke depth is less than 47 mm, the disparities in the principal strain evolution under different friction coefficients are not substantial. Nevertheless, when the punch stroke depth surpasses 47 mm, the smaller the friction coefficient, the more rapidly the principal strain increases. Taking into account the influence of the friction coefficient on the forming limit of TA17 titanium alloy deep drawing, to enhance the stamping forming limit performance of the titanium alloy, the friction between the sheet metal and the die should be minimized.

**Figure 12:**
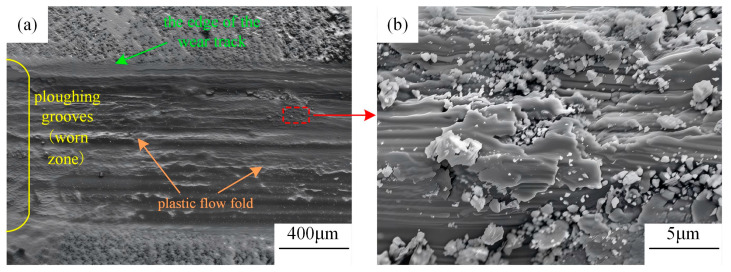
(**a**) Macrotopography of wear trace; (**b**) microtopography of wear trace.

The low-magnification SEM morphology of the worn surface of TA17 titanium alloy after friction test at 700 °C is shown in Figure 12. The worn surface exhibits typical reciprocating sliding wear characteristics, with a wear track width of approximately 2.2 mm. Continuous and parallel ploughing grooves run through the entire wear zone along the sliding direction, which is a typical feature of abrasive wear [26]. The ploughing grooves have uneven depths, and local areas show obvious plastic flow folds, indicating that TA17 titanium alloy undergoes significant softening and plastic deformation at high temperatures, and the material migrates along the sliding direction under the combined action of normal load and tangential friction force [27]. A large number of fine white wear debris particles are scattered on the worn surface. The boundary between the worn zone and the unworn zone is clear, and material accumulation and spalling pits can be observed at the edge of the wear track, which is caused by repeated extrusion and shear fatigue of the material during reciprocating sliding.

This morphology further verifies the composite wear mechanism of TA17 titanium alloy at high temperatures: abrasive wear, adhesive wear and oxidative wear coexist. The ploughing grooves are formed by the scratching of hard abrasive particles, mainly TiO_2_-Al_2_O_3_ oxide products and spalled GCr15 steel debris, on the alloy surface. The plastic flow folds reflect the softening and deformation characteristics of titanium alloy at elevated temperatures. The white wear debris is mainly composed of oxide particles generated by oxidative wear and metal spalls caused by adhesive wear. It is worth noting that no severe adhesive transfer or large-area surface spalling is observed on the worn surface, which confirms that the continuous TiO_2_-Al_2_O_3_ composite oxide film formed at high temperatures effectively isolates the direct contact between the titanium alloy and the steel ball, reduces the degree of adhesive wear, and thus leads to the decrease of friction coefficient with increasing temperature [14].

## 4. Conclusions

In this study, three typical microstructures of TA17 titanium alloy were prepared by different annealing processes. The effects of initial microstructure and friction coefficient on the high-temperature forming performance of TA17 alloy were systematically investigated, and the joint regulatory effect was revealed. The main conclusions are as follows:

1. The initial microstructure has a decisive influence on the high-temperature plasticity of TA17 alloy. The equiaxed microstructure obtained by annealing at 850 °C for 2 h exhibits the best high-temperature plasticity at 900 °C and 0.01 s^−1^, with a fracture mode of microvoid coalescence ductile fracture. The excellent plasticity may be attributed to the effect of grain boundary sliding and dislocation movement promoted by fine equiaxed grains and uniformly dispersed residual β phase. In contrast, the Widmanstätten and bimodal structures show significantly reduced high-temperature plasticity due to coarse grains and lamellar structure-induced stress concentration.
2. The high-temperature friction coefficient of TA17 alloy shows strong temperature dependence. It decreases sharply from 0.56 at 650 °C to 0.19 at 800 °C. The friction coefficient has a significant quantitative influence on the deep-drawing forming limit of TA17 alloy. As the friction coefficient increases from 0.05 to 0.2, the limit principal strain d and limit punch stroke decrease accordingly. Reducing the friction coefficient can effectively improve the material flowability and avoid early fracture caused by stress concentration.

Fine equiaxed microstructure and low friction coefficient are the two key factors to improve the high-temperature formability of TA17 titanium alloy. In actual hot forming processes, TA17 alloy with equiaxed microstructure is recommended. High-temperature lubricants should be adopted to reduce the friction coefficient. As such, desirable forming quality can be achieved. Future work will focus on the development of special high-temperature lubricants for TA17 alloy and the optimization of hot forming process parameters considering the synergistic effect of microstructure and friction.

## Figures and Tables

**Table 1:** The composition of TA17 (obtained from Baoji Titanium Industry Co., Ltd., Baoji, China) [15].

Alloying Element	Al	V	Fe	Si	O	N	H	C	Ti
(wt.%)	4.3	2.1	<0.25	<0.15	<0.15	<0.05	<0.015	<0.08	Bal

**Table 2:** TA17 titanium alloy plate annealing process.

Annealing No.	Annealing Processes
A1	850 °C/2 h/AC (Air cooling)
A2	950 °C/2 h/AC
A3	(950 °C/2 h/AC) + (600 °C/2 h/AC)

**Table 3:** The chemical composition of the ball.

	C	Si	Mn	Cr	P	S
wt.%	0.95–1.05	0.15–0.35	0.20–0.40	1.30–1.60	≤0.027	≤0.02

**Table 4:** High-temperature mechanical properties of TA17 alloy.

Microstructure	Ultimate Tensile Strength (MPa)	Flow Stress at 0.2 Strain (MPa)	Fracture Strain
A1	65.2 ± 1.8	62.1 ± 1.5	1.34 ± 0.04
A2	77.4 ± 2.2	64.8 ± 1.9	0.47 ± 0.03
A3	106.8 ± 2.5	74.9 ± 2.1	0.54 ± 0.03

## Data Availability

The original contributions presented in this study are included in the article. Further inquiries can be directed to the corresponding author.
